# The Complex Role of Mental Time Travel in Depressive and Anxiety Disorders: An Ensemble Perspective

**DOI:** 10.3389/fpsyg.2020.01465

**Published:** 2020-07-21

**Authors:** Ronald T. Kellogg, Cristina A. Chirino, Jeffrey D. Gfeller

**Affiliations:** Department of Psychology, Saint Louis University, St. Louis, MO, United States

**Keywords:** ensemble hypothesis, mental time travel, interpreter bias, executive attention, anxiety, depression

## Abstract

The ensemble hypothesis proposes that uniquely human cognitive abilities depend on more than just language. Besides overt language, inner speech, and causal interpretations, executive attention, mental time travel, and theory of mind abilities are essential parts that combine additively and even multiplicatively. In this review, we consider the implications of the ensemble hypothesis for the psychopathologies of anxiety and depression. Generalized anxiety disorder (GAD) and major depressive disorder (MDD) are two of the most common mental disorders worldwide. The mechanisms that differentiate them are difficult to identify, however. Mental time travel has been implicated in models of depressive and anxiety disorders, but here we argue that at least two other ensemble components, namely, interpreter biases and executive attention, must also be considered. Depressive and anxiety disorders have both been found to show impairments in all three of these components, but the precise relationships seem to distinguish the two kinds of disorders. In reviewing the literature, we develop models for depression and anxiety that take into account an ensemble of mental components that are unique for each disorder. We specify how the relations among mental time travel, interpreter biases, and executive attentional control differ in depression and anxiety. We conclude by considering the implications of these models for treating and conceptualizing anxiety and depression.

## Introduction

Depressive and anxiety disorders are two major categories of psychopathology, yet they have proven difficult to differentiate in some respects. As will be documented below, both are characterized by dysfunctional executive attention and pessimistic attributional styles, with a high degree of comorbidity. Here we build on the premise of Roepke and Seligman (2016) that the core problem in depression is a difficulty in mental time travel, specifically, an inability to envision positive events in the future. We consider the role of mental time travel in differentiating the two disorders and conclude that this component of human cognition is by itself insufficient. Mental time travel, we suggest, is moderated by problems with executive attention and an interpretive component responsible for causal attributions and inner speech.

In an important paper, Roepke and Seligman (2016) argued that prospection, or the mental representation of future events, plays a major role in depression. Human episodic memory enables mental time travel, that is to say, the ability both to recall past autobiographical events and to imagine possible future events (Tulving, 2002). Roepke and Seligman suggested that the negative beliefs about the future and feelings of hopelessness that characterize depressive disorders (Beck, 1974) can be directly linked to faulty prospection, an inability to envision possible futures and poor evaluation of possible futures. In their view, “.faulty prospection is the core causal process of much depression” (p. 24). A similar proposal was advanced by Miloyan et al. (2014) to account for depression; they also extended the analysis by suggesting that a different form of faulty prospection, centered on worry rather than pessimism and hopelessness, lay at the core of anxiety disorders.

We agree that problems with mental time travel are central to psychopathology, but we question whether this component can be isolated from other cognitive components to ascertain its relative contribution. Instead, we argue that other fundamental components of human cognition are concurrently at work in both depressive and anxiety disorders. In our view, it is important to consider how other components impair or even enhance the functioning of mental time travel. To develop this perspective, we draw on the ensemble hypothesis, which holds that human cognition depends on five core systems or components that interact in non-additive ways (Kellogg, 2013; Kellogg and Evans, 2019). Mental time travel is necessary but not sufficient for explaining either the remarkable competencies of human cognition or its breakdowns in disorders such as anxiety and depression. An advanced executive form of working memory, a theory of mind augmenting social cognition, language, the ability to interpret information using inner speech, and causal inference are necessary, as well as an episodic memory capable of mental time travel. Kellogg (2013) introduced the ensemble hypothesis in the context of understanding the exceptional cognitive abilities in the evolution of our species, *Homo sapiens*. The book provides the reasons for considering the five components and their interactions in normally developing and functioning human beings. Kellogg and Evans (2019) offered further evidence in support of the hypothesis from behavioral studies, lesion studies, and studies involving neuro-atypical populations.

The key claim of the ensemble hypothesis is that two or more mental capacities can interact in a multiplicative fashion to yield competencies in a well-functioning human being that exceed their simple additive effects. For example, delay of gratification is a phenomenon that entails an ability both to prospectively consider the future and to exercise cognitive control using executive attention. In typically developing children, growth in the capacity of executive attention for self-regulation boosts the ability to delay rewards in anticipation of a larger future reward (Mischel et al., 1989). Similarly, planning in problem solving requires future thinking and a normally functioning system of executive attention. Frontal lobe injuries that damage networks of executive attention often impair planning (Kellogg and Evans, 2019). In normally functioning adults, retrospective memory for a list of words presented in a laboratory task requires an intact hippocampus and medial temporal lobe, but it is also boosted by maintenance and elaborative rehearsal strategies that depend on executive attention. Failing to deploy attentional resources to an encoding strategy impairs the recall of a list of words presented in a laboratory task in individuals with depression (Hertel and Rude, 1991). As will be considered in detail later, the normal functioning of mental time travel can be altered by depression because of its effects, in part, on executive attention.

The purpose of the present paper is to consider the implications of the ensemble hypothesis for two broad categories of psychopathology: depression and anxiety. We suggest that much of the phenomenology and symptoms that underlie depressive and anxiety disorders can best be understood as an interaction of components of the hypothesized ensemble. We wish to extend the insights provided by Miloyan et al. (2014) and Roepke and Seligman (2016) by demonstrating how the interpreter and executive attention influence mental time travel. As will be seen, language is considered in the form of inner speech, but the broader concept of language as interpersonal communication falls outside the scope of the current paper. Similarly, as will be addressed in the limitation section of our paper, an extensive literature on theory of mind and social cognition in depression ultimately needs to be accounted for. Even so, our focus on the interpreter, executive attention, and mental time travel documents the importance of the interactions posited by the ensemble hypothesis.

**FIGURE 1:**
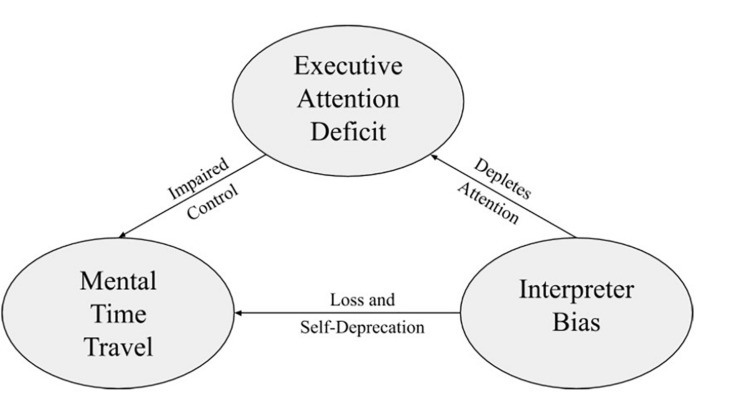
Model of major depressive disorder.

To illustrate, consider the case of depression (see Figure 1), as exemplified by major depressive disorder (MDD). As will be discussed in detail later, the interpreter shown in Figure 1 refers to the inner voice and causal inference capacity of the left hemisphere of the human brain that enables attributions about the self and other people (Gazzaniga, 2000; Kellogg, 2013). In depression, the interpreter is biased to assign blame to the self for negative experiences. This pessimistic and personally negative explanatory style (Petersen and Seligman, 1984) causes the depressed individual to focus attention on negative past events and have difficulty envisioning anything positive about the future. Further, there is evidence that depression is associated with a concurrent deficit in executive attention (Ólafsson et al., 2011), causing impaired cognitive control over mental time travel resulting in persistent negative rumination. Thus, the influence of both a bias in interpretation and a deficit in executive attention, we propose, could underlie faulty prospection in depressed individuals. The interactive model of Figure 1 differs from the position of Roepke and Seligman (2016) with respect to effective approaches to treatment for depression. They advocate for treatments targeting mental time travel, specifically, the core problem with prospection. Alternately, we contend that efforts to improve executive attention and to correct the pessimistic explanatory style of the interpreter ought not be neglected, because they can alter the functioning of mental time travel.

The plan of the paper is, first, to introduce several components of the ensemble hypothesis that are central to our analysis of depressive and anxiety disorders. Second, we consider evidence on the role of mental time travel in depressive and anxiety disorders. Third, we discuss literature regarding the pessimistic explanatory style in depression and suggest that anxious individuals are characterized by a related but distinct dysfunctional style of explaining events as threatening to the self. The emphasis on loss in depression and threats in anxiety can influence the functioning of mental time travel, we propose. Fourth, we document that both kinds of disorders are associated with impairments in executive attention that may compound problems with mental time travel. Fifth, we discuss how the symptoms of depression versus anxiety can best be understood by considering mental time travel, the interpreter, and executive attention as an integrated ensemble. We conclude by considering the implications of the ensemble perspective regarding effective therapies for depressive and anxiety disorders.

## Mental Time Travel, the Interpreter, and Executive Attention

Mental time travel is the unusual form of human episodic memory that allows the mind to recollect the specific time and place of a past event in one’s personal history (Tulving, 2002; Suddendorf and Corballis, 2007). It is conceived as mental time travel because the same neural systems are involved in imagining future events as well as recollecting past events. The brain systems involved in mental time travel include the hippocampus and medial temporal lobe structures as well as the default mode network activated in resting state conditions when no external task is presented (Buckner et al., 2008). The ability to construct spatially coherent scenes in which an event takes place is essential in both recollecting the past and imagining the future. It has been suggested that scene construction is a core function of the hippocampus (Clark and Maguire, 2016).

A uniquely human mental ability appears to be the interpretive capacity of the left hemisphere (Gazzaniga, 2000). Over the course of human evolution, our oral language capacity became internalized as inner speech, mediated by language networks in the left hemisphere. Vygotsky (1962) emphasized that speech begins in early childhood as a means for communication, but as speech is internalized, it also becomes a means for planning and problem solving. Self-directed inner speech, then, has long been recognized as an important vehicle for thinking and appraising situations and events. The interpreter constructs a personal narrative that explains why we feel and behave as we do. Inner speech is combined with a specialization of the left hemisphere for a specific kind of thinking. The left hemisphere is not only specialized for the use of language, including self-directed language of inner speech, but it is also specialized for forming hypotheses (Wolford et al., 2000) and making inferences about causal relationships (Roser et al., 2005). Similarly, the ability to reason deductively is known to be impaired in patients with left frontal lesions but not right frontal lesions (Reverberi et al., 2010).

In clinical psychology, the interpreter is important in understanding the role of inner speech and causal inference in how people respond to stressful life events. How an individual cognitively appraises stressors can either attenuate or exacerbate the strain that they cause. This role for causal attributions has long been recognized in understanding depressive and anxiety disorders. For example, Petersen and Seligman (1984) highlighted that depression is characterized by a personalized and pessimistic explanatory style. The individual attributes personal, pervasive, and permanent causes to negative personal experiences, committing what social psychologists call the fundamental attribution error. The role played by the interpreter in explaining why things happen and what significance events have for the self is central to both depression and anxiety, as will be detailed later in the paper.

The executive attention component of working memory enables the coordination and regulation of representations held in verbal, visual, and spatial stores of short-term memory. Working memory, planning, cognitive control, self-regulation, and response inhibition have all been referred to as executive functions that have traditionally been viewed as dependent on the frontal lobe (Alvarez and Emory, 2006; Posner and Rothbart, 2007; Diamond, 2013; Ajilchi and Nejati, 2017). A more complex understanding has emerged in the literature with two distinct brain networks involved in executive attention; these include but are not limited to regions in the frontal lobe (Posner and Peterson, 1990; Petersen and Posner, 2012).

By studying a battery of executive functioning tasks, Miyake et al. (2000) identified three correlated but distinctive processes underlying performance. Updating the contents of working memory, shifting goals as required in multitasking, and inhibiting irrelevant information are considered three essential and irreducible functions of executive attention. A widely used test of individual differences in working memory capacity, called the Operation Span (OSPAN) test, indicates that the ability to inhibit irrelevant information is especially important and shows a strong correlation with general fluid intelligence or the ability to solve novel problems (Engle et al., 1999).

Mental time travel, the interpreter, and executive attention are three fundamental components of human cognition. Kellogg (2013) proposed that these components, together with theory of mind and language, comprise an ensemble that renders human cognition unique and qualitatively different from non-human cognition. Importantly, his hypothesis suggests that it is the interaction of these components that yields the unique properties of human cognition. If that is so, then it stands to reason that common forms of psychopathology should reveal such interactions, too. In persons experiencing anxiety or depression, a deficit in one component can cascade to degrade the functioning of another component, despite that the latter component is not necessarily dysfunctional.

## Mental Time Travel Impairments

Roepke and Seligman (2016) reviewed a variety of evidence that faulty prospection lies at the heart of depression. First, persons experiencing depression can envision negative future scenarios more readily, compared to non-depressed persons (MacLeod and Byrne, 1996). This characteristic is also shared with those experiencing anxiety, indicating it is not a unique dysfunction of mental time travel associated with depression. Miloyan et al. (2014) suggested that anxious as well as depressed individuals anticipate negative future events but that each disorder shows a unique profile of faulty prospection. Individuals with anxiety anticipate more negative experiences, but not fewer positive experiences, relative to control participants without a history of psychiatric diagnosis, according to some studies (MacLeod and Byrne, 1996; MacLeod et al., 1997b). Depression, on the other hand, is associated with a failure to anticipate positive future events (Miranda and Mennin, 2006; Pomerantz and Rose, 2014). When depressed psychiatric outpatients were asked to describe a distressing personal problem and to imagine and rate the likelihood of both the worst and best possible outcomes, they rated the worst outcome as being more likely and the best outcome as being less likely, relative to generalized anxiety disorder (GAD) and control groups (Beck et al., 2006).

Thus, it is possible that a faulty form of prospection found in depression results in a diminished ability to envision positive future events (MacLeod and Salaminiou, 2001). However, both this finding and the finding that individuals with depression envision more negative future events than do controls can also be linked to a pessimistic explanatory style. MacLeod et al. (1997a) found that both depressed and anxious patients not only judged future negative events to be more likely, relative to controls; they also provided more supportive as opposed to contradictory reasons for their occurrence. As MacLeod et al. (p. 22) concluded, “…mood-disturbed subjects were pessimistic about what would happen to them in the future, and this was supported by their causal thinking about those events.” Thus, the pessimistic explanatory style of the interpreter rather than a malfunction in mental time travel *per se* could explain the findings. They could also be linked to the deficits in executive attention that are associated with depression (Ólafsson et al., 2011). As will be argued in later sections of the paper, problems with mental time travel may arise because of the moderating influences of the interpreter and executive attention.

An important exception regarding memory impairment in depression is the tendency to focus and elaborate upon sad events (Williams et al., 1997). A case can be made for mood congruent memory in depression (Mineka and Nugent, 1995). For example, in a study by Derry and Kuiper (1981), a list of depression-related adjectives (e.g., bleak, dismal, helpless) and non-depression-related adjectives (e.g., amiable, curious, loyal) were presented in an incidental learning task. The nature of the orienting task was manipulated, with one way being whether the adjective applied to the self. On a subsequent recall test, this self-reference orienting task resulted in a greater proportion of depressed-content words recalled (41%) than non-depressed content (16%) for depressed patients. Strikingly, this pattern was completely reversed for normal controls, who recalled more non-depressed content (43%) compared with depressed content (8%). Even a group of psychiatric controls showed a reversal with more non-depressed content (36%) relative to depressed content (18%). None of these effects were observed for structural (small letters?) and semantic (means the same?) orienting tasks, indicating that they are contingent on judging the word as relevant to the self.

Similarly, in another study, after being shown a list of words including pleasant, unpleasant, and neutral words, individuals with depression recalled more unpleasant words compared with pleasant words (McDowall, 1984). A non-depressed control group as well as another control group made up of psychiatric patients with a diagnosis other than depression did not show this bias toward improved memory for unpleasant words. The depressed patients’ free recall of unpleasant words was at the same level as that for the two control groups, whereas they showed a memory impairment for pleasant words. This indicates that the mood congruent benefit of remembering unpleasant words can offset the usual memory impairment found in depression.

Clark and Teasdale (1982) found that autobiographical experiences also reveal mood congruency even within the same group of individuals with depression. The investigators compared the recall of personal memories at two different times of day to capitalize on diurnal variations in mood among psychiatric patients experiencing depression. The percentage of unhappy memories (52.3%) was reliably greater when the individual reported being more depressed compared with less depressed (36.7%). Happy memories (37.7 versus 51.1%) showed exactly the reverse pattern.

The above studies show that depression can bias retrospection in the direction of remembering sad events more readily than happy events. Would such findings also hold for prospection? MacLeod et al. (1997b) measured the recall of past experiences and the anticipation of future experiences in anxious, depressed, and control individuals. The study prompted the participants to remember or anticipate either positive experiences or negative experiences. This prompt variable allowed the comparison of the number of positive events versus negative events produced under conditions of both retrospection and prospection. Their findings showed no difference between the retrospection and prospection conditions for either disorder. Of importance, individuals with depression produced fewer events compared with controls—both positive and negative—both in recalling their past and in anticipating their future.

An analogous outcome has been found in laboratory studies of the retrospective recall of word lists versus prospective memory for future actions. Hertel and Rude (1991) found poorer free recall of a list of words presented earlier for currently depressed patients compared with recovered patients and control individuals with no history of depression in a retrospective task. Rude et al. (1999) similarly reported that depressed individuals perform poorly on a prospective memory task requiring the ability to self-initiate an action in the future. Their difficulties with “remembering to remember” to act in the future were parallel to impairments found in retrospective tasks, according to the authors. Of course, these tasks are different from the autobiographical reports examined by MacLeod et al., but the conclusions reached are consistent. MacLeod et al. (1997a) also found that anxious individuals did not differ from controls either in remembering or in anticipating positive events. However, they generated more negative events compared with controls regardless of whether they were engaged in retrospection or prospection. Their findings thus confirm that anxiety is primarily a disorder of worrying about negative outcomes (Barlow, 1988). Whereas MacLeod et al.’s control participants both recalled and anticipated about 44% more positive life events than negative ones, the anxious participants only recalled 15% more positive events. Compared to participants with depression, the participants with anxiety recalled and anticipated about 67% more negative events.

Finally, MacLeod et al. expected that individuals with depression would show a mood congruent effect by remembering or anticipating more negative events compared with positive events. In contrast to prior studies reviewed earlier, this outcome did not occur. Rather, negative events were remembered by patients with depression at about the same rate as found in the controls. This rate was equivalent to the number of positive events remembered by those with depression, who were 75% less likely to remember positive events than were patients with anxiety and controls. This is reminiscent of the findings with the free recall of word lists reported by McDowall (1984). Unpleasant words were remembered as well by patients with depression as by controls, but recall for pleasant words showed a marked impairment.

The above findings on memory could depend on the severity of the depressive disorder. It is important to note in that regard that MacLeod et al. (1997b) examined patients who met the diagnostic criteria for panic disorder and MDD. Similarly, the studies by Derry and Kuiper (1981), Clark and Teasdale (1982), McDowall (1984), Hertel and Rude (1991), and Rude et al. (1999) examined psychiatric inpatients or patients with depression in the community with screening done to insure they met the diagnostic criterion for depression. By contrast, in a non-clinical student population, neither trait anxiety nor trait depression was associated with difficulties in a measure of prospective memory (Arnold et al., 2014). Thus, the severity of the disorder probably plays a role in the effects of depression and anxiety on mental time travel.

In contrast to the picture for clinical depression, the findings on retrospective memory for anxiety disorders are mixed. MacLeod and McLaughlin (1995) found that individuals currently receiving treatment for GAD performed worse than those in a control group on an explicit recognition test for words presented in a laboratory setting. By contrast, on explicit memory tests of cued recall (Mathews et al., 1989) and free recall (Becker et al., 1999), anxious individuals performed at the same level as control participants. For threatening words included among the lists presented in the laboratory, GAD patients showed no advantage in recall or recognition, but they did show superior performance on various implicit memory tests compared with controls (Mathews et al., 1989; MacLeod and McLaughlin, 1995). A similar heightened explicit memory for threatening words was found by Becker et al. (1999) for individuals diagnosed with panic disorder but not with social phobia or GAD. In a review of the literature on memory and anxiety disorders, Mineka and Nugent (1995) concluded that the evidence for an explicit memory bias for threatening events is weak, difficult to replicate, and unconvincing, at least with respect to persons experiencing GAD.

We conclude from this sample of findings in the literature that while depression impairs mental time travel ability, it does not seem to be a selective difficulty with prospection. Judging from the findings of MacLeod et al. (1997a), at least for positive events, anxious individuals do not appear to show any impairment in mental time travel, either in its prospective or in its retrospective form. In fact, they appear to recollect past negative events and envision future negative events more often than is found in both non-anxious controls and depressed patients. However, other studies indicate that such memory bias for threatening events is tenuous at best in anxiety. Patients with depression, on the other hand, forget positive events more readily than is found in non-depressed controls. A central question is what accounts for these differences in the functioning of the mental time travel component. We propose that considering the role played by the interpreter and executive attention helps to understand the pattern of results found for mental time travel.

## Interpreter Biases

As noted earlier, the interpreter in individuals with depression employs a pessimistic explanatory style (Petersen and Seligman, 1984). An inability to envision a positive future and a facility with envisioning a negative future could be understood as a dysfunction of the explanatory style found in individuals with depression rather than a fault with mental time travel *per se* (MacLeod et al., 1997a). A negative style of explaining why things happen as they do is a prime reason for feelings of hopelessness in depressed people (Alloy et al., 1988). Individuals with depression tend to attribute the reasons for events in life to internalized causes about the self that are stable over time and that are global or pervasive in multiple situations. Another compounding factor is a negative attributional style that attributes negative events to uncontrollable causes (Sanjuán and Magallares, 2009). As a consequence, persons with depression might be able to recollect or imagine an event that most people would regard as positive (e.g., getting a job promotion) but then interpret it as negative. Individuals with depression might appraise the promotion as full of pitfalls—more responsibility, longer working hours, and greater stress. Remembering or anticipating a job promotion may not be the problem but, rather, its pessimistic interpretation.

The interpreter, therefore, has a prominent, if not central, role in depression. Indeed, Beck (1974) designed cognitive behavioral therapy (CBT) to confront and modify a depressed person’s inner speech of hopelessness and self-deprecation. By altering the person’s cognitive appraisals of situations and causal explanations of events, mood improves as a result. Beck’s concept of the cognitive triad included a negative view of the self, negative interpretations of ongoing experiences, and a negative view of the future. Although the latter could be caused by faulty prospection, the first two stem from the distorted explanations of the interpreter.

Comparisons of the interpretative style of depressed versus anxious individuals have yielded conflicting results, however. For example, Heimberg et al. (1989), by contrast, found that the attributional style found in the learned helplessness of individuals with depression was also characteristic of multiple anxiety disorders, such as social phobia, agoraphobia, and panic disorder. In their study, the two disorders differed only in that depression produced global and unstable attributions regarding the causes of positive events, whereas anxiety was associated with the same kind of attributions for negative events. Still other findings indicate that attributing internal, stable, and global causes to negative events is in fact found in currently depressed individuals, but especially in those with comorbid anxiety (Fresco et al., 2006). Similarly, Luten et al. (1997) concluded that a pessimistic attributional style is not specific to depression but, rather, is correlated with high levels of negative affect as is also found in in persons with anxiety disorders. Ahrens and Haaga (1993) even reported that a negative event attributional style was only found with anxiety disorders rather than with depression.

Thus, it seems that pessimistic forms of causal inference about life’s events are a non-specific risk factor for anxiety and depression. This commonality with respect to the dysfunction of the interpreter is a likely reason why depressive and anxiety disorders share a high degree of comorbidity (Gotlib, 1984; Kessler et al., 2007; Grisanzio et al., 2018).

Despite their similarities and high rates of comorbidity, there may be some unique aspects to the interpreter’s dysfunction in anxiety disorders, however. Riskind and Williams (2005) identified a looming cognitive style in which individuals overestimate the progression of a potential threat in terms of both spatial and temporal dimensions. Individuals with a high score on their looming cognitive scale misinterpret potential threats as catastrophic threats. A study by Reardon and Williams (2007) showed that this looming cognitive style is uniquely associated with anxiety disorders. A pessimistic cognitive style contributed to both anxiety disorders and depressive disorders, but individuals predisposed to anxiety disorders also were prone to a looming cognitive style that magnifies potential threats. Anxiety disorders also feature highly persistent negative self-talk. The excessive worry that characterizes anxiety is largely verbal in nature (Borkovec et al., 1998). Instead of imagining a threat in a visual–spatial context, anxious individuals talk to themselves about it. Finally, it has long been recognized that the causal inferences made in depression are associated with personal failures and self-deprecation (Beck, 1974). This contrasts with worries about uncertainties and potential dangers in the case of anxiety disorders (Beck et al., 1987; Clark et al., 1990).

**FIGURE 2:**
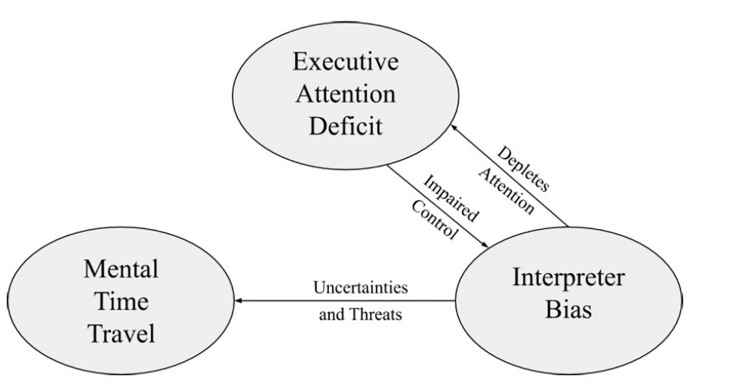
Model of generalized anxiety disorder.

As shown in Figure 2, the anxious interpreter views events as threatening to the self rather than as a negative reflection of the self as in depression (Figure 1). Kendall and Ingram (1989) differentiated the two disorders precisely in terms of their characteristic attributions. The interpretations of the depressive person often are “self-referent, definitive, past-oriented cognitions of sadness, failure, degradation, and loss,” in contrast to the “future oriented ‘questioning’ cognitions” found in anxiety disorders (Kendall and Ingram, 1989; p. 36).

In our view, the interpreter biases play a role in the problems observed in mental time travel with depressed and anxious individuals (Eysenck et al., 2006). For depression (Figure 1), the pessimism of the interpreter causes individuals with depression to remember and ruminate about negative life experiences that reinforce feelings of loss and self-blame. Perhaps the difficulty with imagining a positive future is a direct consequence of depressed individuals focusing on negative past events. Roepke and Seligman (2016, p. 27), in fact, suggest the possibility that persons experiencing depression “struggle to recall a good past,” with few positive memories (Williams and Scott, 1988).

For GAD (Figure 2), the interpreter is biased to detect threats to the self. This might cause one to recall, see, and foresee dangers rather than losses. Instead of mental time travel being impaired relative to control individuals who are neither depressed nor anxious, there is, if anything, an excessive prospection and retrospection of negative events. This outcome can be seen in the study by MacLeod et al. (1997b), who reported that anxious individuals both retrospectively and prospectively generated more negative events than did controls and even individuals with depression. Similarly, when asked to recall recent life events, 77% of anxious participants remembered danger events compared to 44% of depressed participants (Finlay-Jones and Brown, 1981). Loss events, on the other hand, were more frequently remembered by participants with depression (65%) compared with anxiety (15%). Comparable findings for autobiographical recall have been reported by Witheridge et al. (2010).

Thus, in our view, the interpreter plays a key role in the functioning of the mental time travel component. The content of the events that are remembered in depression is more likely to deal with loss rather than danger. This, we suggest, occurs because of the bias of the interpreter on mental time travel. In anxiety disorders, retrospection is not impaired; if anything, there is excessive rather than impaired prospection and retrospection about threatening events. In addition to mental time travel being moderated by the interpreter, we further consider in the next section the possible influence of executive attention.

## Executive Attention Deficits in Depression and Anxiety

A number of studies have shown that anxiety and depression are associated with impaired performance on a variety of neuropsychological tests that measure for executive control functions (Reinholdt-Dunne et al., 2013; Devito et al., 2018). This supports the notion that both disorders are associated with impairments in executive attentional control. In this article, we refer to executive attention as executive attentional control and attentional control, interchangeably. In accordance with Stefanopoulou et al. (2014, p. 330), attentional control can be defined as “the ability to sustain focus on tasks in the face of competing activities or to shift attention from one task to another.” However, depression and anxiety do not show the same pattern of executive attention deficits.

The Attentional Control Scale (ACS) is a self-reported attention control measure that is comprised of two components: focusing and shifting (Reinholdt-Dunne et al., 2013). Ólafsson et al. (2011, p.77) define attentional focusing as “the capacity to intentionally hold the attentional focus on desired channels and thereby resist unintentional shifting to irrelevant or distracting channels” and define attentional shifting as “the capacity to intentionally shift the attentional focus to desired channels, thereby avoiding unintentional focusing on particular channels.” Because it has been noted that those with anxiety show attentional impairment in relation to shifting and focusing (Devito et al., 2018), the ACS has been used to compare the relationship between attentional focusing, attentional shifting, and levels of anxiety and depression in adults. Ólafsson et al. (2011) found that when controlled for depression, the focusing ACS subscale significantly predicted anxiety ratings, whereas when anxiety ratings were controlled for, the shifting subscale significantly predicted depression ratings. Reinholdt-Dunne et al. (2013) supported these findings when they found ACS focusing to be associated with lower anxiety and ACS shifting to be associated with fewer depression symptoms. These findings support the claim that anxiety is more associated with attentional focusing and depression is more associated with attentional shifting.

Shi et al. (2019) performed a meta-analysis to investigate the size and nature of attentional control deficits in participants with anxiety versus non-anxious participants. They found that anxiety-producing deficits were supported in processing efficiency, rather than effectiveness, on a variety of behavioral tasks. However, they also found that when looking at task switching studies alone, both efficiency and effectiveness produced anxiety-related deficits in attentional control. Their results also showed that studies requiring participants to operate under high cognitive load conditions showed greater anxiety-related attentional control deficits compared to studies where participants were under normal cognitive load conditions (Shi et al., 2019).

Although attentional control deficits have been related to anxiety disorders, these deficits are prominently seen in individuals diagnosed with GAD, characterized by uncontrollable worry. This uncontrollable worry has been connected to deficits of the central executive function of working memory, which includes attentional control as a key component of working memory (Stefanopoulou et al., 2014). Uncontrollable worrying can be attention-demanding and, consequently, consumes voluntary attentional resources required (Eysenck et al., 2007). This links uncontrollable worry to impairments in attentional control.

Stefanopoulou et al. (2014) used the key-pressing task to assess the extent to which attentional resources were depleted by worry in individuals with GAD. Stefanopoulou et al. (2014) found that GAD individuals were less random on the key-pressing task while worrying compared to when thinking of a positive topic, indicating that fewer residual attentional control resources were available during the worrying process. However, the performance of the healthy participants did not differ between conditions. GAD participants also reported having more negative thoughts and anxiety during this task compared to healthy participants. This same study also used the N-back task, which “varies in difficulty and is sensitive to subtle difference in ability to handle increasing demands on attentional control” (Stefanopoulou et al., 2014, p. 330). During this task, GAD participants exhibited longer reaction times compared to healthy participants for the higher load conditions. These results together indicate a greater difficulty in sustaining focus in conditions requiring a higher degree of attentional control, suggesting that poor attentional control may partially explain the excessive worry seen in individuals with GAD.

Further, there appears to be a bidirectional relationship between attentional control and anxiety (Devito et al., 2018). Impairments in attentional control may increase one’s risk for developing anxiety, and anxiety symptoms may prevent executive components of attention from being recruited. We indicate this bidirectional relationship between the interpreter and executive attention in Figure 2. The pessimistic explanatory style and negative self-talk of the interpreter consume limited attentional resources. The resulting deficit in executive attention weakens the ability to inhibit the dysfunctional thinking of the interpreter in anxiety disorders.

Whether a similar bidirectional relationship occurs in depression is unclear. An argument against this takes into account the speech and inner speech of depressed versus anxious individuals based on the symptoms outlined in the *Diagnostic and Statistical Manual of Mental Disorders, Fifth Edition* (*DSM-V*). The hallmark of GAD is excessive worry in the form of inner speech. By contrast, in MDD, fatigue and tiredness occur on nearly a daily basis, and this can be accompanied by slowed speech, long pauses before responding, and a decrease in the amount and variety of speech content (American Psychiatric Association, 2013, p. 132). These suggest that inner speech in MDD is more likely to be inhibited or overly regulated rather than exaggerated, as is apparent in GAD. Moreover, in a review of the literature on inner speech, Alderson-Day and Fernyhough (2015) noted that the evidence for inner speech playing a central role in anxiety disorders is stronger and more specific than it is with depression. The verbalized worry of anxiety is, in their words (p. 948), “…repetitive thinking that is.negative, uncontrollable, and aimed at some ill-defined problem solving, such as a problem with a clear solution.” We propose that the bidirectional links between executive attention and the interpreter produce worry in GAD that is indeed out of control (see Figure 2). A positive feedback loop ensues in which worry depletes attention, which in turn worsens worry. In depression, the negative impact of depleting attention does not appear to feed back on the interpreter. Instead, we suggest, it feeds forward to impact mental time travel. Specifically, the deficit in executive attention found in depression results in a loss of control in mental time travel (see Figure 1). The arrows shown in Figures 1, 2 are intended to reflect the major pathways of influence from one component to another. From the perspective of the ensemble hypothesis, all possible links among components are potentially relevant, including bidirectional relationships. In a normally developed and well-functioning adult human being, each of these components influences the others. Our aim in these figures is to take a minimalist approach by highlighting only strong interactions that differ from normal under a diagnosis of psychopathology. The purpose is to differentiate as clearly as possible how MDD and GAD differ from each other. For example, we intentionally omit an influence of executive attention on mental time travel in GAD. Although it is known that the availability of executive attention affects the functioning of mental time travel even in healthy individuals, we only indicate interactions that are unique to GAD or MDD.

## Memory Impairment From an Ensemble Perspective

As shown in Figure 1, we suggest that both retrospection and prospection will be impaired as a result of a deficit in executive attention (Hertel and Rude, 1991; Rude et al., 1999). Evidence for a causal role played by attention comes from an intervention designed by Hertel and Rude (1991) to remediate the attentional deficits. Hertel and Rude studied three groups of individuals who were currently depressed, recovered from depression, or without a history of depression in an incidental learning and memory task. The participants’ ability to recall a list of target words that they had viewed in the first phase of the experiment was markedly impaired in the individuals with depression compared with recovered and healthy controls. But this outcome only occurred when their attention to the words during learning was unconstrained by the demands of the task. For half of the participants, the investigators required the participants to repeat the target words aloud on each trial, as a means of focusing their attention. Strikingly, this manipulation eliminated the memory impairment of the depressed patients entirely. This result suggests that retrospection *per se* is not necessarily deficient in depression, but a memory deficit can be observed as a result of the influence of executive attention not being appropriately allocated to the task at hand.

A comparable finding was reported by McDowall (1984). On a free recall test, inpatients with depression performed markedly worse than did a control group consisting of non-depressed psychiatric inpatients in remembering pleasant words. However, when given an orienting task of rating each word for pleasantness as was shown during the study phase, patients with depression showed no difference in recall between pleasant and unpleasant words and performed no worse than did the psychiatric control group doing the same task. Their mean recall of 5.6 words out of 12 was only slightly less than was found for a non-psychiatric control group (6.8 words), again with no difference between pleasant versus unpleasant words. As with the word repetition technique used by Hertel and Rude (1991), the orienting task directed attention to the words in a way that eliminated most, if not all, of the memory impairment for individuals with depression.

Ruminating on negative life experiences is part and parcel of the sense of loss, hopelessness, and self-deprecation frequently seen in persons experiencing depression. In our view, these phenomena are the direct result of the interpreter bias found in depression. It is the influence of the interpreter with mental time travel that contributes to the inability of individuals with depression to think about positive life experiences, whether they lie in the past, the present, or the future. Further, the persistence and intrusiveness of negative memories in depression could reflect an inability to inhibit them because of executive attention deficits (see Figure 1). Poor cognitive control may combine with the loss bias of the interpreter to produce the profile of memory problems found in depression.

As shown in Figure 2, for GAD, the mental time travel system is biased to focus on the uncertainties and threats of life experiences. Instead of loss and self-blame, the content of memories predominately concerns threats to the self in anxiety disorders to the extent that they are biased at all. This can account for why negative events are, at times, better remembered or anticipated by anxious individuals (e.g., MacLeod et al., 1997b). However, in contrast to the memory bias effects for losses observed in depression, similar effects for threatening events in anxiety disorders are harder to detect reliably (Mineka and Nugent, 1995). They might be found in panic disorder but not GAD (Becker et al., 1999). Or they can be observed with implicit memory tests but not explicit tests of recall or recognition (Mathews et al., 1989; MacLeod and McLaughlin, 1995). They might also be observed when people are asked to recall autobiographical events of personal relevance (Finlay-Jones and Brown, 1981; Witheridge et al., 2010) but not when they are asked to remember word lists that contain some threatening versus neutral words (Levy and Mineka, 1998).

We suggest that the mixed picture for memory bias in anxiety disorders occurs because executive attention deficits do not generally disrupt mental time travel in persons experiencing GAD, which is not the case for MDD (see Figure 2). The deficit in executive attention causes a loss of control with the interpreter but not with mental time travel. Without both a loss of cognitive control and a threat bias from the interpreter, the mental time travel system functions relatively normally in GAD. That implicit tests of memory reveal bias effects for negative information implies that a threat bias from the interpreter is at work. But for the declarative memory system of episodic memory to show such effects, it requires both the threat bias and a loss of cognitive control over mental time travel. Perhaps only in severe cases of anxiety disorders, such as panic disorder, does the loss of cognitive control from deficits in executive attention spill over to affect mental time travel, much as it does in depression. This could account for the results of Becker et al. (1999) for panic disorder in contrast with other forms of anxiety disorder. It is worth noting that MacLeod et al. (1997b) studied anxious participants who all met the criteria for panic disorder. Thus, the characteristics of their sample might have explained why they observed a bias for negative events when so many other studies have been unable to do so, as they noted in their discussion section.

In summary, accounting for the consistent memory bias for losses or a lack of positive memories in MDD seems to depend on distorting inputs from both executive attention and the interpreter (see Figure 1). For persons experiencing GAD without panic disorder, the input from executive attention is weak or non-existent. Without this concomitant symptomatology, the bias of the interpreter for threatening events does not distort either retrospective or prospective memory, although it shows up on implicit, non-declarative forms of memory.

## Limitations, Implications, and Future Directions

As noted previously, our explication of the complex role of mental time travel in explaining the phenomenology and research findings related to MDD and GAD has focused on interrelationships between three of the five components of the ensemble hypothesis. In focusing on these three constructs, we acknowledge the limited attention we have given to the importance of the two remaining ensemble components—overt use of language and social cognition—in accounting for differences and similarities in MDD and GAD. Reviewing the broader concept of language as interpersonal communication falls outside the scope of the current paper. Similarly, the extensive literature on theory of mind and social cognition in disorders such as MDD and GAD merits careful consideration that is not undertaken by our current analysis. Research indicates that theory of mind, a specialized aspect of social cognition (Frith and Frith, 2007), plays a complex role in presentations of depression and anxiety where aspects of social cognition are prominent (Bora and Berk, 2016; Washburn et al., 2016). Examples would include depression in the context of discordant relationships or bereavement, and social anxiety disorder. Exploring the interrelationships between social cognition and other components of the ensemble hypothesis is a fruitful direction for further theorizing and research.

Also, our paper is limited in scope, in that we focused on accounting for differences between disorders such as MDD and GAD, rather than examining similarities in their phenomenology and accounting for the high comorbidity of these conditions. We believe that further analysis of the interrelationships among the ensemble of mental components in MDD and GAD may help account for the comorbidity of these two disorders. For example, the high incidence of comorbidity might be accounted for by the reciprocal relations between the cognitive ensemble components and symptoms that constitute pathways that connect the disorders (Borsboom and Cramer, 2013). It is worth noting the strong similarities of MDD and GAD as portrayed in Figures 1, 2. Both disorders involve several components of the ensemble hypothesis, including executive attention and the interpreter, in addition to mental time travel. The specific characteristics of memory functioning seem to depend on these interrelated cognitive components of the ensemble perspective. Thus, future theorizing and research should explore the interrelated components of the ensemble hypothesis as they relate to comorbid presentations of MDD and GAD.

Regarding one final limitation of our paper, we acknowledge that the ensemble component of “mental time travel” as it pertains to episodic foresight involves multiple constructs, each with substantive theoretical and empirical literatures that lie beyond the scope of our paper. Examples would include the role of mental time travel in future decision making involving delayed rewards (Boyer, 2008) and the literature on “affective forecasting” (Wilson and Gilbert, 2005) as it relates to the ensemble components in persons experiencing depression or anxiety. Once again, future theorizing and research should explore the interrelationships of such constructs with the ensemble components as they pertain to the etiology and phenomenology of MDD and GAD.

In review, we believe that similarities and differences between MDD and GAD are best conceptualized by considering an ensemble of mental components. Although mental time travel plays a role in both disorders, this component is influenced by the interpreter that assigns causal attributions to events and a dysfunction in executive attention.

If depression is primarily a problem with faulty prospection, then it is reasonable to target future thinking as perhaps the most effective form of treatment. Roepke and Seligman (2016) reviewed four variations of CBT that emphasize positive expectancies, hopeful thinking, a focus on future-oriented solutions to problems, and goal setting and planning. Initial results with each of these approaches have been positive and are worthy of additional study in randomized trials. Further, Roepke and Seligman (2016) suggest several new future-oriented interventions that might be considered (e.g., using visual imagery to imagine a route to future success).

While new approaches certainly merit exploration, we note that the premise underlying these—namely, that faulty prospection is the core causal process in depression—is open to debate. We believe that the effects of the interpreter and executive attention, in conjunction with mental time travel, should be considered to better understand both MDD (Figure 1) and GAD (Figure 2). From this ensemble perspective, therapies should target all three components rather than focusing only on mental time travel.

For example, mindfulness-based therapies including short-term meditation explicitly address deficits in executive attention. A short-term program (5 days of training for 20 min per day) has been shown to improve attention and self-regulation in a sample of healthy young adults (Tang et al., 2007). Such mindfulness-based interventions have been shown to minimize relapse and offer promise in the treatment of acute symptoms of depression and anxiety, although more research is needed to clearly establish their clinical efficacy (Edenfield and Saeed, 2012). In a different approach, training attention using computer-based tasks has been found beneficial in treating generalized social anxiety disorder (Schmidt et al., 2009). If anxiety disorders as well as depression primarily are influenced by the mental time travel component (Miloyan et al., 2014), then it is difficult to explain why treatments targeting the executive attention deficit would be effective. Yet, it is known that executive functioning matters. Although neurocognitive abilities can improve with CBT treatment for anxiety and depression, individuals with poor attentional control show decreased benefit from such treatment compared to those with adequate executive skills (Devito et al., 2018).

Many techniques in traditional CBT build on the premise of altering the pessimistic explanatory styles employed by depressive and anxious individuals. These techniques are based on the premise that the symptoms and dysfunctional behaviors of these disorders are mediated by cognitive factors. The therapeutic goal, then, is to restructure the dysfunctional thinking and beliefs underlying the disorder. Cognitive distortions must be identified and refuted in restructuring the functions of the interpreter. The evidence supporting CBT as an effective treatment of both anxiety and depression is solid (Butler et al., 2006). As Roepke and Seligman (2016) pointed out, CBT interventions already include a number of techniques that improve future thinking. Even so, the aim of CBT is to alter thinking patterns in general, including past and present thinking as well as future thinking. It is not clear that new approaches that emphasize future-oriented thinking only would be, or even should be, superior to standard CBT.

In terms of future directions, transdiagnostic psychotherapies for depression and anxiety (Clark, 2009) could potentially be understood within and informed by the aspects of the ensemble hypothesis. The ensemble models shown in Figures 1, 2 suggest that a unified approach to CBT plus mindfulness/attention training might well be plausible for treating both depressive and anxiety disorders. Finally, in recent years, network approaches to psychopathology have emphasized the interplay of symptoms across a variety of traditionally defined, yet comorbid, disorders (Borsboom and Cramer, 2013). The psychopathology network approach contends that such emotional disorders arise from interactions among symptoms, as well as their reciprocally reinforcing relationships (Borsboom, 2017). It may be possible to conceptualize these networks of psychopathology within the context of the ensemble hypothesis of human cognition considered here.

## Data Availability Statement

The original contributions presented in the study are included in the article/supplementary material. Further inquiries can be directed to the corresponding author.

## Author Contributions

RK developed the concept of the paper. RK and CC wrote the first draft. JG contributed with advice and revisions to subsequent drafts. All authors reviewed the final manuscript.

## Conflict of Interest

The authors declare that the research was conducted in the absence of any commercial or financial relationships that could be construed as a potential conflict of interest.
